# Radium and War Wounds

**Published:** 1918-12

**Authors:** 


**Radium and War-wounds.** By Dr. Simon Laborde. *Revue Interalliée pour les Mutilés de la Guerre,* April, 1918.

A special service for the treatment by radium of vicious scars resulting from war-wounds was organized in August, 1915, in the military hospital of the Grand-Palais.

Cases treated in that way are of many kinds: defective scars, cicatricial adherences, atonic wounds, and dermatitis developed around those wounds.

Four sorts of apparatus are used:

(*a*) Sealed tubes containing radium salts; (*b*) sealed ampules containing radium emanation; these are very practical, for they provide powerful radiation in a convenient form; (*c*) flat apparatus covered with radiating enamel; (*d*) an apparatus distributing radio-active water for irrigation of wounds.

The tubes contain 12 to 25 mgr. of radium metal. The salts included in them are either sulphate or bromide.

The ampules have at first a charge of 100 millicuries. Emanation is filtered so that only the $\gamma$ and most rapid $\beta$ rays can pass through. Tissues are protected against the action of the secondary rays by sheathing the tubes with india-rubber and gauze. No inflammation of the skin has ever been observed.

Applications last from one to twenty-four hours, or more, according to the cases.

*Defective Scars.* A defective scar may paralyze a peripheral nerve or be the cause of painful neuritis. In these different cases applications of radium, and also X-ray action, have often resulted in considerable improvement. In the beginning, the treatment comports per week 3 applications of 2 hours each with 25 mgr. of radium metal, filtrated by 0.5 mm. of platinum. Complete cures have been obtained in this way after 4 or 5 weeks. If after this time only a slight improvement appears, 3 applications lasting each 15 or 20 hours, and separated by 10 days intervals, are tried.

The action of radium on *sore stumps* is very marked. It often renders possible the keeping on of prothesis apparatus that could not be supported before. Generally 3 applications of 24 hours each have proved to be sufficient.

The *painful scars* are healed or greatly improved in 85/100 of the cases.

A defective scar may also be the cause of *muscular adherences*, hindering or limiting certain movements. Radium has often proved active in such cases, especially if the scar and adherences are still recent.

*Atonic Wounds.* Wounds sometimes 18 months or 2 years old have healed in two or three weeks under the action of radium emanation. They are irrigated for 10 minutes every day with radio-active water, and a wet dressing of this water is kept upon them. On an average 2/3 of the cases heal.

*Eczemas — Dermatitis.* Complete healings may also be obtained in those cases, even in ancient and severe ones.

